# Rectal Diverticulum as a Rare Complication Following the Longo Procedure: A Case Report and Review of Therapeutic Challenges

**DOI:** 10.7759/cureus.82513

**Published:** 2025-04-18

**Authors:** Arturs Niedritis, Sergejs Lebedjkovs

**Affiliations:** 1 Department of General Surgery, Riga Stradins University, Riga, LVA; 2 Department of General Surgery, AIWA Clinic, Riga, LVA

**Keywords:** hemorrhoid surgery, longo procedure, rectal diverticulum, rectocele, stapled hemorrhoidopexy, surgical complications

## Abstract

Iatrogenic rectal diverticulum is an exceptionally rare complication following the Longo procedure (stapled hemorrhoidopexy), with only a handful of cases reported in the literature. This case is noteworthy due to its rarity, the diagnostic challenges it presents, and its therapeutic implications for patients undergoing stapled hemorrhoidopexy. Reporting this case aims to raise awareness among clinicians about this uncommon complication and contribute to the understanding of its clinical management.

A 60-year-old woman underwent the Longo procedure in 2021 for symptomatic Grade III hemorrhoidal disease and rectocele. The initial surgery was successful, with no immediate postoperative complications. However, four years later, she presented with rectal discomfort but no other significant symptoms. Diagnostic evaluation, including colonoscopy and rectal ultrasonography, revealed a large diverticulum at the site of the previous stapled anastomosis, at five o'clock position. Surgical intervention was planned, involving resection of the diverticulum and primary repair of the rectal wall. Histopathological examination confirmed the presence of a true diverticulum involving all layers of the rectal wall. The patient recovered well postoperatively, with resolution of symptoms and no further complications at follow-up.

This case highlights rectal diverticulum as a rare but significant late complication of the Longo procedure. It underscores the importance of considering this entity in patients with persistent or recurrent symptoms after stapled hemorrhoidopexy. Long-term follow-up and a high index of suspicion are essential for timely diagnosis and management. The case also emphasizes the need for further research to understand the underlying mechanisms, such as altered rectal wall mechanics or stapling-related tissue changes, and to optimize treatment strategies. Clinicians should be aware of this complication to improve patient outcomes and guide informed decision-making in the management of hemorrhoidal disease.

## Introduction

The Longo procedure, or stapled hemorrhoidopexy, is a widely used surgical technique for the treatment of symptomatic internal hemorrhoids and rectocele. Introduced in the late 1990s, it offers advantages such as reduced postoperative pain and shorter recovery times compared to traditional hemorrhoidectomy [1,2]. However, like any surgical procedure, it is not without complications. Common adverse events include bleeding, pain, fecal urgency, and recurrence of hemorrhoids, while rare complications such as rectal perforation, pelvic sepsis, and stenosis have also been reported [3,4].

Among the rarest complications is the development of a rectal diverticulum, a condition characterized by the herniation of rectal wall layers. Only a few cases of rectal diverticulum following the Longo procedure have been documented in the literature, making it a poorly understood entity [5,6]. According to current literature, rectal diverticulosis accounts for 0.1% of cases of colonic diverticulosis [7], while the incidence of pseudodiverticula may reach 2.5% [8]. The pathogenesis is thought to involve altered rectal wall mechanics, ischemia at the stapling site, or technical factors during surgery, but the exact mechanism remains unclear [9,10]. If diagnosed in an untimely matter this complication may lead to patient discomfort, as mentioned in this case, obstructive defecation syndrome, potential fistula formation and due to the infective nature of diverticular pouch, possible abscess formation. Yet the information on this subject is limited.

This case report describes a 60-year-old woman who developed a rectal diverticulum four years after undergoing the Longo procedure, presenting a diagnostic and therapeutic challenge. The rarity of this complication and the lack of standardized management guidelines underscore the importance of reporting such cases. This report aims to contribute to the limited body of literature on rectal diverticulum post-Longo procedure, highlight its clinical implications, and provide insights into its management.

## Case presentation

A 60-year-old woman presented to our clinic in 2025 with a four-month history of rectal discomfort and intermittent anal pain. The patient reported the ability to palpate a solid mass through the vagina. She had undergone the Longo procedure (stapled hemorrhoidopexy) in 2021 for symptomatic Grade III hemorrhoidal disease and rectocele. The procedure was successful, with no immediate postoperative complications.

On physical examination, digital rectal palpation revealed a soft, tender mass approximately 6 cm from the anal verge and about 4 cm above the dentate line. Rectoscopy demonstrated a rectal diverticulum measuring approximately 6 × 3 cm at the 5 o’clock position, containing a firm intraluminal mass. A colonoscopy performed two years earlier for an unrelated indication showed no pathological findings. Routine gynecological ultrasonography also revealed no abnormalities. It was decided to operate the patient due to patient reported discomfort and potentially dangerous infectious process. Both the initial procedure and the subsequent reoperation were performed by the same board-certified surgeon.

**Figure 1:**
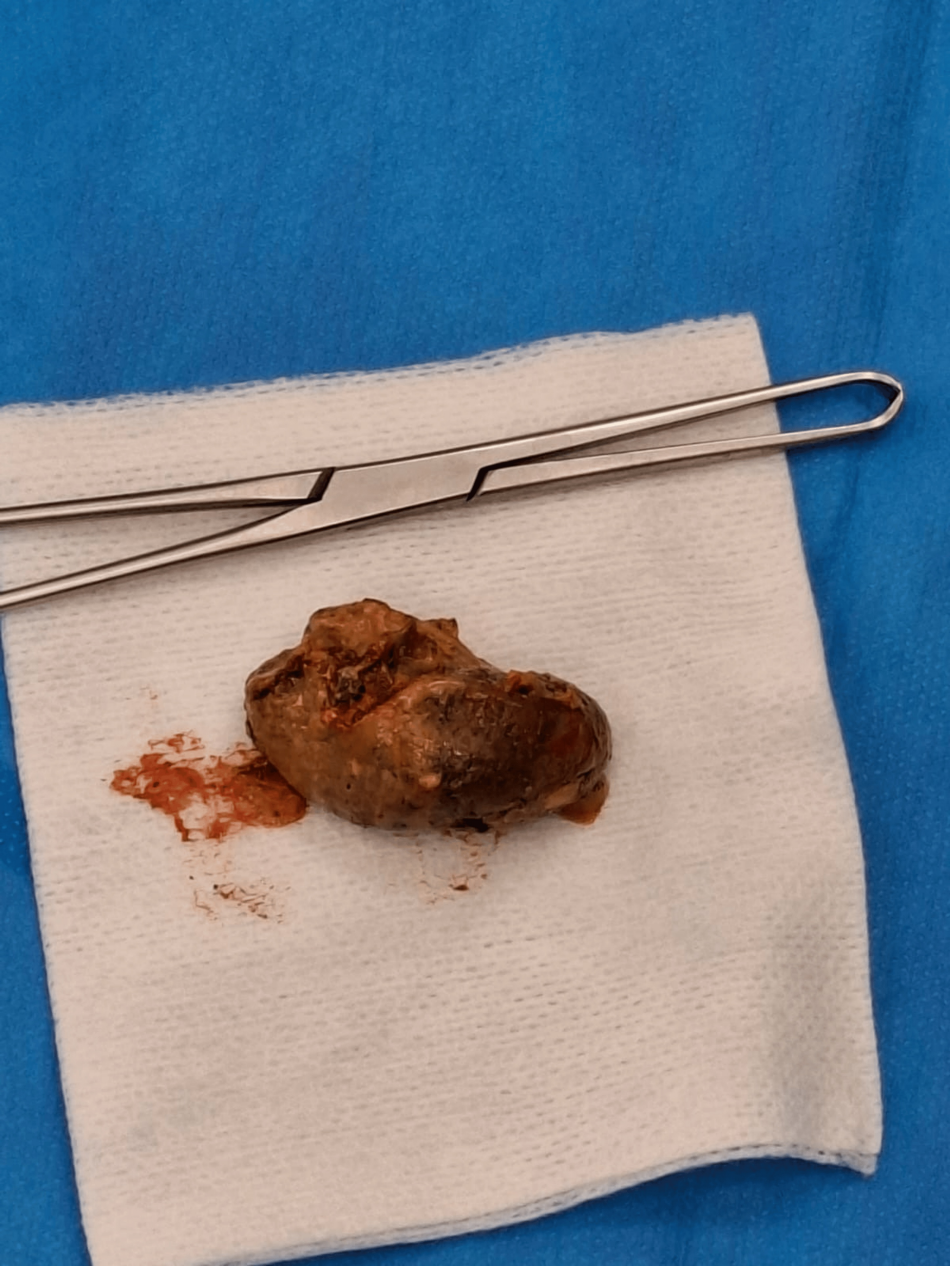
Coprolite embedded into the rectal diverticulum The image shows a large coprolite embedded in the rectal diverticulum, which the patient was able to palpate and was most likely the cause of her symptoms.

Prior to reoperation, rectal preparation was achieved with a sodium citrate/ sodium lauryl sulfoacetate/ glycerol suppository. Transanal diverticuloectomy was planned. The patient underwent surgical intervention under general anesthesia. Operation was performed transrectally. Plastic, size 34 anal mirror was used to visualize operating field. Careful dissection of the diverticulum was performed using a harmonic scalpel, ensuring preservation of the surrounding muscle layers to minimize functional impairment. The coprolite inside the diverticulum was removed, to make sure that complete diverticulum excision is achieved and diverticulum was everted after which the diverticulum was excised (Figure 1).

**Figure 2:**
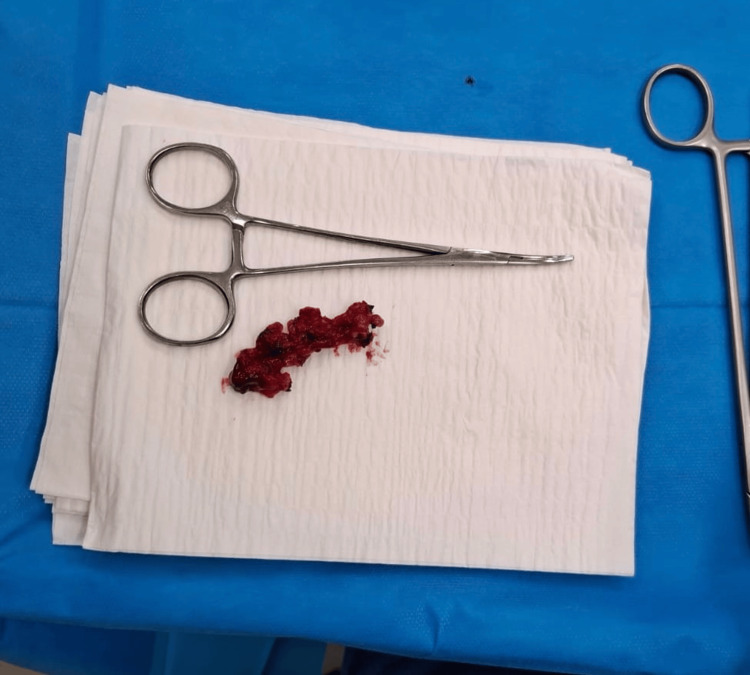
Excised diverticular tissue

Due to the presence of pus and fecal contamination, the defect was closed with absorbable interrupted 3-0 Novosyn Polyglactin-910 sutures (B. Braun, Melsungen, Germany) to reduce the risk of infection and promote healing. Excision borders were adapted to ensure free passage of feces and prevent accumulation in the area of defect, sutures were also applied in a manner to allow for free drainage. Hemostasis was meticulously achieved, and the surgical site was irrigated thoroughly. The excised tissue was sent for histopathological examination, which confirmed the presence of a true diverticulum involving all layers of the rectal wall, with evidence of chronic inflammation and fibrosis (Figure 2). No staples were identified macroscopically. After the operation, a hemostatic sponge was inserted into the anal canal.

Postoperatively, the patient was managed with broad-spectrum antibiotics, specifically metronidazole 250 mg three times a day for seven days, as well as analgesics, including paracetamol 500 mg twice daily as needed for pain. Additionally, she received lactulose 10-20 mL once daily before bed for two months. She recovered well, with no immediate complications, and was discharged on the first postoperative day after thorough physical examination, making sure that the patient is in stable clinical condition, has no pain or bleeding, successfully passes stool, and lacks systemic signs of infection, tolerates food, also the patient was instructed to immediately contact the clinic if any discomfort, bleeding, difficulty urinating or defecating, or other potential complications arise. At follow-up visits every two weeks for two months, she reported complete resolution of her symptoms, and no recurrence was observed on transrectal ultrasonography.

This case highlights the technical challenges of managing rectal diverticulum as a rare complication of the Longo procedure, emphasizing the importance of meticulous surgical technique and postoperative care to ensure optimal outcomes.

## Discussion

Rectal diverticulum following the Longo procedure (stapled hemorrhoidopexy) is an exceptionally rare complication. In our center's experience, approximately 0.16% of cases have developed true or pseudodiverticula. This case contributes to the limited body of knowledge on this unusual entity and underscores the importance of considering it in patients with persistent or recurrent symptoms after stapled hemorrhoidopexy. The development of a rectal diverticulum in this patient, four years after the initial surgery, raises intriguing questions about its pathogenesis and the long-term implications of the Longo procedure.

The exact mechanism behind the formation of rectal diverticula after stapled hemorrhoidopexy remains speculative. One possible explanation is the alteration in rectal wall mechanics caused by the stapling device. The circular stapler creates a mechanical anastomosis, which may lead to localised ischemia, weakening of the rectal wall, and subsequent herniation of the mucosa and submucosa through the muscular layer [5,9]. Additionally, the presence of chronic inflammation or infection at the stapling site, as seen in this case, could further compromise tissue integrity and contribute to diverticulum formation [4]. Another potential factor is the technical execution of the procedure, such as excessive tension during stapling or improper placement of the device, which may predispose the patient to tissue damage and subsequent complications [3].

The possibility of developing symptomatic rectal pseudodiverticula as a long-term complication of failed purse-string sutures during stapled hemorrhoidopexy, also known as rectal pocket syndrome, is an important consideration [10]. Incomplete or improperly placed purse-string sutures can create pockets or pseudodiverticula that accumulate fecal material, leading to chronic inflammation or infection. This mechanism may also explain the development of true diverticula, as seen in our case, where chronic inflammation and mechanical stress could lead to full-thickness herniation of the rectal wall.

The incidence of rectal diverticula is exceedingly low, with estimates suggesting they occur in only 0.1% of colonic diverticula cases [7]. Symptomatic cases are even rarer, making large-scale studies challenging. However, a systematic review which analyzed 78 studies encompassing 14,232 patients who underwent stapled hemorrhoidopexy, reported overall complication rates ranging from 3.3% to 81%, including five mortalities [11]. However, this review did not detail specific data on rectal diverticula, underscoring the rarity of the condition and the need for heightened clinical awareness. 

Our performed intervention- transanal diverticuloectomy provided adequate result in our case. Yet other methods are also possible, but the approach should be individualised to each patient, for example, transabdominal mesh rectopexy, conservative management, radiological intervention. It is advised to operate these diverticula in symptomatic patients. Transanal approach is also recommended in cases of diagnostic uncertainty [9]. The patient was discharged on first post-operative day after physical assessment of the patient, it is not the standard practice in full-thickness rectal excisions, yet it is a possibility in correctly selected patients [12].

Proper purse-string suture placement is critical in stapled hemorrhoidopexy to ensure optimal tissue approximation and reduce the risk of complications such as rectal pocket syndrome and diverticulum formation. The suture should be placed approximately 4 cm above the dentate line in a circumferential manner, incorporating only the mucosa and submucosa while avoiding excessive tension that could lead to tissue strangulation or ischemia. Uniform spacing is essential to prevent gaps or asymmetric closure, which may predispose to incomplete tissue incorporation during stapling. Additionally, ensuring adequate rectal eversion before firing the stapler helps to minimize the risk of residual pockets or herniation. It is of utmost importance to examine the rectal doughnut after excision to ensure that it encompasses the entire circumference without gaps and each patient should be evaluated for potential necessity of staple line reinforcement with absorbable sutures. Meticulous attention to these technical details can significantly reduce the incidence of postoperative complications and improve surgical outcomes. It is important to conduct further studies on the prevalence of this complication following Longo procedure, as it is one of the most often performed operations for hemorrhoidal disease. It is imperative to understand the underlying mechanism of post-operative rectal diverticula development and potential connection to incorrect surgical technique and to ensure that this operation is performed in correctly selected patients.

## Conclusions

True rectal diverticulum represents a rare but clinically significant complication of the Longo procedure, with potential implications for patient outcomes and surgical practice. This case underscores the importance of understanding the underlying mechanisms, refining surgical techniques, and maintaining vigilant follow-up to ensure timely diagnosis and management. Most importantly intraoperative assessment of purse-string suture and staple line for uniformity and correct spacing should be done during operation. Further research is needed to explore the pathophysiology of these complications and to develop evidence-based guidelines for their prevention and treatment.
